# THE PREDICTIVE EFFECT OF DIFFERENT MACHINE LEARNING ALGORITHMS FOR PRESSURE INJURIES: A NETWORK META-ANALYSES

**DOI:** 10.1093/geroni/igad104.3776

**Published:** 2023-12-21

**Authors:** Chaoran Qu, Xiufen Yang, Weisi Peng, Xiujuan Wang, Weixiang Luo

**Affiliations:** Shenzhen People’s Hospital, Shenzhen, Guangdong, China (People’s Republic); Shenzhen People’s Hospital, Shenzhen, Guangdong, China (People’s Republic); Shenzhen People’s Hospital, Shenzhen, Guangdong, China (People’s Republic); Shenzhen People’s Hospital, Shenzhen, Guangdong, China (People’s Republic); Shenzhen People’s Hospital, Shenzhen, Guangdong, China (People’s Republic)

## Abstract

**Objective:**

This review aims to systematically synthesize existing evidence to determine the effectiveness of applying machine learning algorithms for pressure injury management, to further evaluate and compare pressure injury prediction models constructed by numerous machine learning algorithms, and to derive evidence for the best algorithms for predicting and managing pressure injuries.

**Methods:**

A systematic electronic search was conducted in the EBSCO, Embase, PubMed, and Web of Science databases. We included all retrospective diagnostic accuracy trials and prospective diagnostic accuracy trials constructing a predictive model by machine learning for pressure injuries up to December 2021. The network meta-analysis was conducted using statistical software R and STATA. The certainty of the evidence was rated using the QUADAS-2 tool.

**Result:**

Twenty-five clinical diagnostic trials with a total of 237397 participants were identified in this review. The results of our study revealed that pressure injury machine learning models can effectively predict these injuries. Combining the algorithms separately yields the main

**results:**

decision trees (sensitivity: 0.66, specificity: 0.90, AUC: 0.88), logistic regression (sensitivity: 0.71, specificity: 0.83, AUC: 0.84), neural networks (sensitivity: 0.73, specificity: 0.78, AUC: 0.82), random forests (sensitivity: 0.72, specificity: 0.96, AUC: 0.95), support vector machines (sensitivity: 0.81, specificity: 0.81, AUC: 0.88). According to the analysis of ROC and AUC values, random forest is the best algorithm for the prediction model of pressure injury.

**Conclusions:**

This review revealed that machine learning algorithms are generally effective in predicting pressure injuries, the random forest algorithm is the best algorithm for pressure injury prediction.

